# The Emerging Mechanisms of Wnt Secretion and Signaling in Development

**DOI:** 10.3389/fcell.2021.714746

**Published:** 2021-08-16

**Authors:** Shefali Mehta, Swapnil Hingole, Varun Chaudhary

**Affiliations:** Department of Biological Sciences, Indian Institute of Science Education and Research Bhopal, Bhopal, India

**Keywords:** Wnt, wingless, morphogen gradient, frizzled, secretion, signaling

## Abstract

Wnts are highly-conserved lipid-modified secreted proteins that activate multiple signaling pathways. These pathways regulate crucial processes during various stages of development and maintain tissue homeostasis in adults. One of the most fascinating aspects of Wnt protein is that despite being hydrophobic, they are known to travel several cell distances in the extracellular space. Research on Wnts in the past four decades has identified several factors and uncovered mechanisms regulating their expression, secretion, and mode of extracellular travel. More recently, analyses on the importance of Wnt protein gradients in the growth and patterning of developing tissues have recognized the complex interplay of signaling mechanisms that help in maintaining tissue homeostasis. This review aims to present an overview of the evidence for the various modes of Wnt protein secretion and signaling and discuss mechanisms providing precision and robustness to the developing tissues.

## Introduction

Wnt proteins are secreted signaling molecules present in all metazoans. Signaling pathways activated by Wnt proteins play a crucial role in governing various aspects of development, including cell fate determination, body axis patterning, cell migration, cell proliferation, tissue maintenance and tissue regeneration (Logan and Nusse, 2004; Steinhart and Angers, 2018). Dysregulation of Wnt signaling pathways leads to developmental disorders such as bone density defects (Gong et al., 2001; Little et al., 2002; Ugur and Tolun, 2008; Baron and Kneissel, 2013), defective stem cells homeostasis (Ring et al., 2014; Nalapareddy et al., 2017) and progression of several diseases such as colorectal, pancreatic and breast cancers (Nusse and Clevers, 2017; Zhan et al., 2017). Hence, Wnt signaling has been a focus of intensive investigation over decades in the area of biomedical research.

Analysis of Wnt-mediated processes during development have revealed Wnt proteins to possess morphogen-like activity. According to the classical definition, morphogens are diffusible molecules, which form a gradient across a field of cells and activate the target gene expression in a concentration-dependent manner, thereby establishing tissue patterns via differential gene expression (Turing, 1952; Wolpert, 1969, 1971; Gierer and Meinhardt, 1972). However, Wnt proteins are hydrophobic in nature due to lipid-modification (Willert et al., 2003; Takada et al., 2006; Janda et al., 2012). This creates an intriguing problem to understand their mode of travel in the aqueous extracellular space. Studies on Wnt proteins in recent years have made considerable advancements in uncovering: (1) the intracellular route of Wnt protein trafficking, (2) the possible modes via which Wnt proteins travel in the extracellular environment, (3) the mechanisms of signaling in developing tissues, (4) the feedback mechanisms modulating pathway activation levels. Our current understanding of these processes is built upon the pioneering work on genetically amenable model organisms, such as *Drosophila melanogaster*. Here, we aim to review the progress made in understanding the mechanisms of Wnt protein secretion, spreading and the mechanisms of signaling in developing tissues.

## Wnt Proteins and Their Discovery

Identification of developmental phenotypes associated with Wnts preceded the characterization of their gene sequences and loci. Initially, the pioneering work of T. H. Morgan in *Drosophila* led to the identification of an X-ray-induced dominant mutation called *Glazed*, which showed a narrow and smooth eyed phenotype (Morgan et al., 1936). Several decades later, an ethyl methanesulfonate (EMS) induced mutant called *wingless^1^ (wg^1^)* was identified, which as the name suggests, showed a loss of adult *Drosophila* wing structures (Sharma, 1973; Sharma and Chopra, 1976). Subsequently, other loss-of-function alleles of *wg* were also identified in a large-scale mutagenesis screen that showed early embryonic patterning defects and lethality (Nüsslein-Volhard and Wieschaus, 1980; Baker, 1987). Complementation analysis and cloning of the *wg* locus linked all the mutations to the same gene, where *wg*^1^ was shown to be a loss-of-function deletion in the 3′ UTR of the *wg* gene (Baker, 1987; Van den Heuvel et al., 1993; Schubiger et al., 2010), whereas *Glazed* was shown to be a gain-of-function mutation caused due to the insertion of a retrotransposon upstream of the *wg* gene (Brunner et al., 1999). The vertebrate homolog of the *Drosophila wg* gene was initially identified in mice as an oncogenic integration site for a retrovirus- mouse mammary tumor virus (MMTV) called *int-1* (Nusse and Varmus, 1982; Rijsewijk et al., 1987) and was later renamed as *wnt* (Nusse et al., 1991). Further analysis of the *wnt* (*int1)* encoded protein showed a cysteine-rich domain (CRD) and a signal sequence, indicating that Wnts are secreted proteins (van Ooyen and Nusse, 1984; Fung et al., 1985). Since then, several members of the Wnt family have been discovered across the metazoans. For instance, to date, 19 different mammalian and 7 different *Drosophila* Wnt proteins have been identified (Wnt homepage^1^), all of which share a common signature of 23-24 highly conserved cysteine residues at the N-terminus of their peptide (Nusse and Varmus, 1992).

## Overview of Wnt Signaling

**FIGURE 1:**
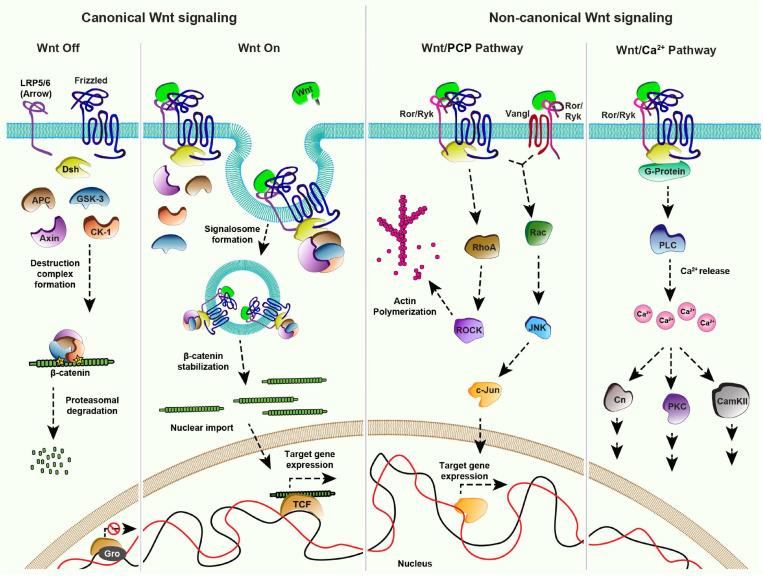
Wnt signaling pathways: **Canonical Signaling- Wnt-Off**: In absence of the ligand (Wnt), the receptor (Frizzled/Fz) and co-receptor LRP5/6 (Arrow) remain in an inactive state at the plasma membrane. In the cytoplasm, components of the destruction complex (GSK-3β, APC, CK-1, Axin) bind and phosphorylate the nuclear effector β-catenin, followed by its ubiquitination and proteasomal degradation. In the nucleus, transcriptional repressor Groucho (Gro) binds to the co-factor TCF and keeps Wnt target gene expression off. **Canonical signaling- Wnt-On:** Binding of Wnt to the Fz receptor recruits LRP5/6 forming an active receptor complex, which leads to Dishevelled (Dsh)-mediated inactivation of the destruction complex. The consequential accumulation of β-catenin in the cytoplasm enables its nuclear import. The nuclear β-catenin replaces Gro from TCF forming a transcriptional activator complex leading to Wnt target gene expression. **Non-canonical Wnt/PCP pathway:** The binding of Wnt to the Fz receptor leads to the recruitment of Dsh. This complex along with the co-receptors, for example, receptor tyrosine kinase-like orphan receptor (Ror) and tyrosine-protein kinase (Ryk) can activate effector kinases like RhoA/ROCK, which leads to actin polymerization. Wnt proteins can also induce the activation of the c-Jun N-terminal kinase (JNK) pathway through both Fz-Dsh-Ror/Ryk and Vangl-Ror/Ryk protein complexes. **Non-canonical Wnt/Ca^2 +^ pathway:** Fz receptor recruits the co-receptors Ror/Ryk upon binding of Wnt ligands which activates Dsh and G-proteins (α,β) at the membrane forming an active cluster. This results in the activation of phospholipase-C (PLC) leading to the release of intracellular calcium ions. Increased calcium levels further activate different pathways mediated by downstream effectors namely Calcineurin (Cn), Calmodulin dependent protein Kinase II (CAMK II) and Protein Kinase C (PKC).

Once released by the producing cells, Wnt proteins travel in the extracellular space, to reach their target cells by mechanisms discussed below. In the target cells, Wnt proteins activate signaling by interacting with the extracellular CRD of the cell surface GPCR receptors called Frizzled (Fz) (Bhanot et al., 1996; Strutt et al., 2012). However, in many organisms the complexity of signal activation increases significantly due to the presence of multiple Fz receptors, interacting with a repertoire of Wnt ligands (Niehrs, 2012). A combinatorial interaction of different Wnt proteins with Fz receptors leads to the activation of multiple downstream pathways (Figure 1), which are classified into two broad categories based on the involvement of β-catenin: the β-catenin dependent canonical Wnt pathway and the β-catenin independent non-canonical Wnt pathways.

The canonical Wnt pathway is activated by the stabilization and nuclear import of β-catenin which leads to the expression of Wnt target genes. Several components of the Wnt/β-catenin signaling pathway have been identified by studies using powerful genetic model organisms (Jenny and Basler, 2014). It is now well-established that in the Wnt-Off state (Figure 1), levels of β-catenin are kept low in the cytoplasm by a “destruction complex” comprising of serine-threonine kinases, Glycogen synthase kinase 3 (GSK3 also known as Zeste-white 3), Casein kinase α1 (CK1), scaffolding proteins Axin and Adenomatous Polyposis Coli (APC) (Siegfried et al., 1990, 1992; Peifer et al., 1994a,b; Liu et al., 2002). Once phosphorylated by GSK3 and CK1, β-catenin is ubiquitinated and degraded via proteasome (Aberle et al., 1997; Kitagawa et al., 1999). Activation of the canonical signaling pathway by the binding of Wnt ligands to the Fz receptors further recruits the co-receptors LRP5/6 (Arrow in *Drosophila*) (Bhanot et al., 1996; Cadigan et al., 1998; Zhang and Carthew, 1998; Tamai et al., 2000; Wehrli et al., 2000; Dann et al., 2001; Piddini, 2005; Figure 1). These receptor complexes aggregate at the membrane to form a signalosome by the recruitment of a cytoplasmic protein Dishevelled (Dsh) to the membrane (Bilic et al., 2007; Gammons et al., 2016). This in turn sequesters the destruction complex to the plasma membrane resulting in β-catenin stabilization followed by its nuclear import and activation of Wnt target genes (van de Wetering et al., 1997).

Wnt proteins can also activate pathways independent of β-catenin which are categorized as non-canonical Wnt signaling pathways. One of the best-studied examples of non-canonical Wnt signaling is the planar cell polarity (PCP) pathway. Initially identified in *Drosophila*, the PCP pathway consists of six core components; Fz, Dsh, Prickle (Pk), Strabismus/Van Gogh (Vang) or Vang-like (Vangl) in vertebrates, Flamingo (Fmi) and Diego (Dgo), which are conserved amongst the metazoans (Eaton, 1997; Seifert and Mlodzik, 2007; Hale and Strutt, 2015; Butler and Wallingford, 2017; Humphries and Mlodzik, 2018). In various tissues, the pathway is governed by asymmetric localization of the core components as two separate complexes consisting of Vang-Fmi-Pk and Fz-Fmi-Dsh-Dgo (Usui et al., 1999; Axelrod, 2001; Feiguin et al., 2001; Shimada et al., 2001; Strutt, 2001; Tree et al., 2002; Bastock et al., 2003), at the opposite ends of the cell (reviewed in Harrison et al., 2020). These complexes activate downstream signaling which determines various polarized cellular outputs such as cell shape regulation, the orientation of primary cilia in the vertebrate inner ear, directed cell migration and the directional organization of tissues (Yang and Mlodzik, 2015; Humphries and Mlodzik, 2018). For example, the receptor complex initiates the Rho family GTPase cascade leading to the cytoskeletal rearrangements (Strutt et al., 1997; Winter et al., 2001; Marlow et al., 2002; Tanegashima et al., 2008). Moreover, the receptor complex, along with the co-receptors of receptor tyrosine kinase family (Ror or Ryk) can also activate c-Jun N-terminal kinase (JNK) signaling via Rac1, which further leads to c-Jun/activator protein 1 (AP1)-mediated expression of the JNK target genes (Eaton et al., 1995; Boutros et al., 1998; Fanto et al., 2000; Oishi et al., 2003; Schambony and Wedlich, 2007; Green et al., 2014; Figure 1).

The role of Wnt proteins in the regulation of PCP has been studied in several model organisms. For example, gradients of Wnt proteins have been shown to regulate global polarization of PCP in the developing vertebrate tissues (Parr et al., 1993; Yamaguchi et al., 1999; Fisher et al., 2008; Gao et al., 2011, 2018; Chu and Sokol, 2016; Minegishi et al., 2017) and providing directional cues for the elongation of myocytes in chicken (Gros et al., 2009). However, while the function of the PCP pathway in tissue patterning is well-established in *Drosophila*, the role of Wnt ligands in the activation of PCP remains controversial. A previous study showed that localized mis-expression of Wnts in the developing fly wing epithelium can modulate the global orientation of the cellular asymmetry (Wu et al., 2013). However, in contrast, two independent recent studies showed that removal of endogenous Wnts did not affect the PCP pathway (Ewen-Campen et al., 2020; Yu et al., 2020).

Another important non-canonical pathway is the Wnt/Ca^2+^pathway. In this case, the binding of Wnt ligands to the Fz receptors and co-receptor Ror/Ryk leads to G-protein-mediated activation of phospholipase C (Figure 1). This further leads to an increase in the intracellular Ca^2+^concentration and concomitant activation of the Calmodulin-dependent kinase or protein kinase C (PKC) pathway (Kühl et al., 2000; Kohn and Moon, 2005). Wnt/Ca^2+^ pathway is involved in several developmental processes such as ventral fate determination in *Xenopus* embryos and axonal guidance in mammals (De, 2011; Ng et al., 2019).

## Synthesis and Intracellular Transport of Wnt Proteins

**FIGURE 2:**
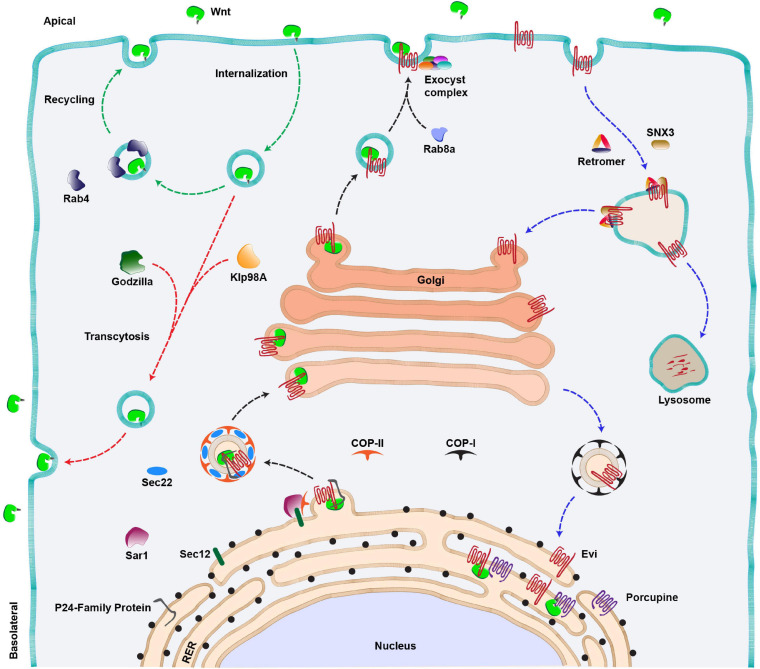
Wnt secretion pathway. Anterograde route of Wnt secretion (black arrows): Newly synthesized Wnt proteins are transferred to the lumen of RER where they are palmitoylated by an acyltransferase (Porcupine). This is followed by the binding of lipid-modified Wnts to their cargo receptor, Evenness interrupted (Evi). The Wnt-Evi complex is then transported from ER to Golgi by Sec22/COP-II vesicles, aided by various members of the P24 family of proteins, Sec12 and Sar1 proteins. Golgi to membrane transport of Wnt protein is mediated by Rab8a and a multi-protein exocyst complex further mediates the apical release of Wnts from the polarized epithelial cells. Wnt proteins can also be internalized and the internalized Wnts are either recycled and secreted apically via Rab4 endosomes (green arrows) or transcytosed (red arrow) to the basolateral side of the producing cell via an E3 ubiquitin ligase (Godzilla). The kinesin motor Klp98A is also involved in apical to basolateral transcytosis of Wnts. Retrograde route of Evi trafficking (Blue arrows): The Wnt-unbound Evi is transported back to the Golgi and RER with the help of retromer complex/SNX3 and COP-I vesicle, respectively. In the absence of the retromer function, Evi is degraded in the lysosomes.

Post translation, all the Wnt proteins, except for *Drosophila* WntD (Wnt inhibitor of Dorsal), are lipid-modified in the lumen of the endoplasmic reticulum (ER) (Figure 2). This occurs via palmitoylation by an ER-membrane-bound-O-acyl-transferase called Porcupine (Van den Heuvel et al., 1993; Kadowaki et al., 1996; Willert et al., 2003; Figure 2). Earlier studies reported two palmitoylation sites on Wnt proteins at a conserved serine and a cysteine residue (Willert et al., 2003; Takada et al., 2006; Janda et al., 2012). However, structural analysis of *Xenopus* Wnt8, by a later study, showed that Wnt proteins are mono-palmitoylated at the conserved serine residue, whereas the conserved cysteine residue is involved in a disulfide bond (Willert et al., 2003; Takada et al., 2006; Janda et al., 2012). Palmitoylation is essential for the secretion of Wnt proteins, and therefore, mutation in the conserved serine residue or loss of Porcupine activity leads to retention of Wnt proteins in the ER (Takada et al., 2006; Barrott et al., 2011; Biechele et al., 2011). Besides lipidation, Wnt proteins are also glycosylated at several residues (Smolich et al., 1993). The pattern of glycosylation varies between different Wnt proteins, which was shown to regulate apical or basolateral sorting of Wnt protein in polarized cells (Yamamoto et al., 2013). However, unlike palmitoylation, the role of glycosylation is poorly understood.

Following the transport and modification of Wnt proteins in the ER, they are transferred to a cargo-receptor protein Wntless (also known as Sprinter/Evenness interrupted (Evi)/MIG-14/Gpr177, referred to as Evi hereafter), a multipass transmembrane protein which is an essential component of the Wnt secretory pathway (Bartscherer et al., 2006; Bänziger et al., 2006; Goodman et al., 2006). Recent structural analysis of the Wnt-Evi complex revealed that post palmitoylation, Wnt proteins are loaded on Evi through direct lateral transfer from Porcupine (Nygaard et al., 2021). Furthermore, the lipidation of Wnt protein is also essential for binding with Evi (Coombs et al., 2010; Herr and Basler, 2012). This provides an understanding of why defects in palmitoylation cause Wnts to accumulate in the ER.

The next step in Wnt protein secretion requires members of the highly conserved p24 family proteins, which mediate the transport of cargo from ER to Golgi via COPII coated vesicles (Castillon et al., 2011). For example, CHOp24, Eclair, Opm, p24-1 proteins were identified from two independent Wg secretion-related RNAi screens in cultured *Drosophila* cells (Buechling et al., 2011; Port et al., 2011). A later study identified another member of this family, Baiser, which was shown to be involved in Wg secretion (Li et al., 2015). Baiser is suggested to form a complex with a conserved v-SNARE, Sec22 which aids in the fusion of Wg containing vesicles with t-SNARE on the Golgi membrane (Lewis et al., 1997). Besides this, the Wnt-Evi complex has been shown to interact with Sec12 and Sar1 proteins in the ER, which further assist in the formation of COPII vesicles (Sun et al., 2017). Altogether, these studies suggest that the exit of Wnt proteins from the ER follows a tightly regulated pathway requiring specific proteins. The route taken by the Wnt-Evi complex beyond Golgi is poorly understood. However, studies using human cell lines and mouse intestinal Paneth cells have identified Rab8a as a regulator of post-Golgi transport of Wnt proteins to the cell membrane (Figure 2, black arrow) (Das et al., 2015).

After the anterograde transport of Wnts to the membrane, it is believed that the Wnt-unbound Evi is internalized via the clathrin-dependent pathway, and it is further recycled back to the trans-Golgi network (TGN). The retrieval of Evi from endosomes to the TGN is mediated by retromer complex proteins VPS26, VPS29 and VPS35 (Coudreuse et al., 2006; Prasad and Clark, 2006; Belenkaya et al., 2008; Franch-Marro et al., 2008; Pan et al., 2008; Port et al., 2008; Yang et al., 2008) and the associated sortin nexin SNX3 (Harterink et al., 2011b; Zhang et al., 2011) (Figure 2, blue arrows). In the absence of retromer, Evi protein is routed to lysosomes which leads to its degradation and concomitant loss of Wnt protein secretion. Studies in mammalian cells have shown that the retrograde movement of Evi brings it to the ER where it interacts with Porcupine and lipidated Wnts to continue the next rounds of secretion (Yu et al., 2014).

An important and largely unresolved question in the process of Wnt secretion is how and where Wnt proteins separate from Evi. Studies on murine Wnt3a have shown that acidification of secretory vesicles facilitates the dissociation of the Wnt-Evi complex (Coombs et al., 2010; Herr and Basler, 2012). However, interestingly, Wnt proteins are also internalized by the producing cells (Pfeiffer et al., 2002; Yamazaki et al., 2016), and both Evi and Wnts are found in the late endosomal compartments (Gross et al., 2012). Furthermore, it is believed that internalized Wnt proteins are recycled back to the membrane for secretion (Pfeiffer et al., 2002; Yamazaki et al., 2016; Linnemannstöns et al., 2020; Witte et al., 2020; Figure 2, red and green arrows). Therefore, whether Evi and Wnt proteins separate during the anterograde route or if they are internalized together and the separation occurs during the retrograde or the recycling route is unclear and requires deeper analysis of the process.

## Wnt Secretion and Signaling Mechanisms in Polarized Cells

Wnt proteins also play an important role in the development of tissues made of polarized epithelial cells. Generation of a functional extracellular pool of the ligand by these cells requires polarized secretion, either from the apical or basolateral side. Studies have shown that Wnt proteins take a specific route for secretion from the polarized cells, which is believed to regulate their signaling abilities. For example, in polarized Madin–Darby canine kidney (MDCK) epithelial cells Wnt11 and Wnt3a are secreted preferentially from the apical or basolateral side, respectively. This polarized sorting of Wnt11 and Wnt3a is decided by their differential glycosylation patterns and the complexity of the glycans (Yamamoto et al., 2013). Interestingly, it was also shown that while both Wnt11 and Wnt3a needed Evi to reach the Golgi complex, post-Golgi trafficking of Wnt3a to the basolateral side, but not the apical trafficking of Wnt11, appears to be Evi mediated. While the functional significance of differential polarized secretion of Wnt11 and Wnt3a remained unclear, this study highlights the fact that polarized secretion of Wnt ligands is regulated by multiple mechanisms. It will be interesting to further explore these mechanisms in other organisms where polarized cells produce multiple species of Wnt ligands.

Other important examples of polarized Wnt secretion are from studies in *Drosophila* epithelial cells. For instance, analysis of Wg in *Drosophila* embryonic epidermal cells showed that both *wg* mRNA and Wg protein are localized apically (González et al., 1991; Strigini and Cohen, 2000; Simmonds et al., 2001; Pfeiffer et al., 2002). Mis-localization of *wg* mRNA causes defects in Wg secretion and signaling, suggesting that apical secretion is required for the proper functioning of Wg in the embryonic epidermis. Apart from this, the development of *Drosophila* wing epithelium, which mostly contains tightly packed columnar epithelial cells (Fristrom and Fristrom, 1993), is regulated by Wg expressed by a narrow strip of cells at the dorsal-ventral boundary. Similar to the embryonic epidermis, the intracellular Wg protein is localized apically in the Wg producing cells of wing discs. At the extracellular levels, a broad gradient of Wg is observed mostly at the basolateral side (Strigini and Cohen, 2000; Simmonds et al., 2001), however, a short-range extracellular Wg is also reported at the apical side of the columnar cells (Gallet et al., 2008). Which of these secretion routes is required for proper signaling in the receiving cells is debatable. On one hand, a study, using Wg fusion protein, showed that the basolateral secretion of Wg occurs via apical-to-basolateral transcytosis in the producing cells (Figure 2, red arrows). This process requires an E3 ubiquitin ligase called Godzilla, which upon removal showed a reduction in the Wg target gene expression (Yamazaki et al., 2016). However, whether Godzilla is also required for Wg signal transduction in the receiving cells is not known. On the other hand, the rescue of Wg secretion defects in *evi* homozygous mutants by a pulse of Evi expression via a transgene showed that Wg was predominantly released apically by the newly synthesized Evi protein (Chaudhary and Boutros, 2019). It was also shown that the apical secretion of Wg was mediated via an octameric exocyst complex (Figure 2, black dashed line) and the apically secreted pool of Wg is functionally highly active (Chaudhary and Boutros, 2019). Moreover, a recent study has also shown that the apically internalized Wg could be recycled back to the apical side in a Rab4-dependent manner (Figure 2, green arrows) and that the apical to basolateral transcytosis via the kinesin motor Klp98A may not be essential for high-level signaling (Linnemannstöns et al., 2020; Witte et al., 2020; Figure 2, red arrows).

An important question is whether the differential signaling abilities of the apical and basolateral pool of Wg are determined by the mechanisms in the receiving cells or the properties of the ligand? Studies have shown that Wg is internalized largely from the apical side of the receiving cells. These apically-derived Wg containing vesicles are believed to fuse with the Fz2-containing vesicles internalized from the basolateral side, leading to ligand-receptor interaction and activation of the pathway (Marois et al., 2006; Gallet et al., 2008; Hemalatha et al., 2016). Besides this, the Fz1 receptor, which is redundant with Fz2 for canonical signaling is also transported apically (Strutt, 2001; Wu et al., 2004), however, if the Wg and Fz1 interaction also require similar internalization remains unknown. Furthermore, other members of the signaling pathway, for example, the co-receptor Arrow and Wnt-binding protein Dlp are also transported apically (Marois et al., 2006; Gallet et al., 2008; Hemalatha et al., 2016). Thus, the differences in signaling abilities of apical and basolateral Wg may be due to the polarized localization of signaling components.

However, another possibility is that the signaling variations in Wnt ligands are due to their association with different interacting molecules, which may be specifically released from the apical and basolateral sides. These Wnt-binding molecules, besides aiding in the release and spreading of hydrophobic Wnts (discussed further below), may also bestow different signaling abilities to the Wnt ligands.

## Modes of Extracellular Wnt Transport

**FIGURE 3:**
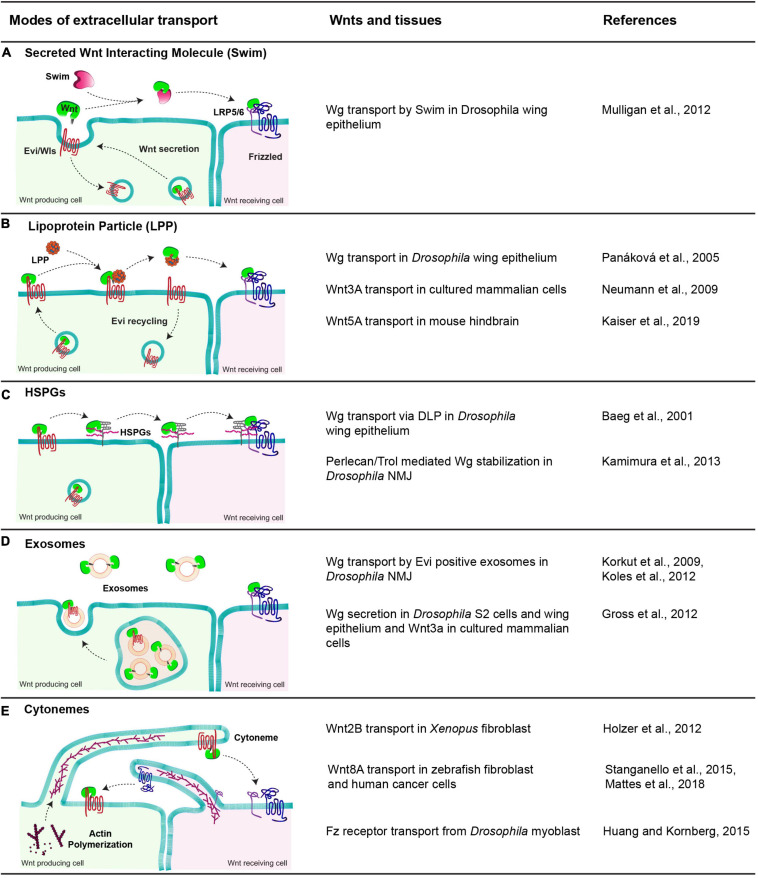
Modes of extracellular Wnt transport. The table shows a diagrammatic representation of different modes employed to facilitate the extracellular travel of hydrophobic Wnt proteins and the tissues these mechanisms are observed. **(A)** Swim, an extracellular carrier protein that binds secreted Wg in *Drosophila* wing epithelium and enables their transport to the receiving cells. **(B)** Lipoprotein particle, a complex of proteins, phospholipids and fats. Wnts are transferred to lipoproteins from Evi, which increases the solubility of Wnts in the extracellular space and allows them to reach the receiving cells. **(C)** HSPGs, these membrane-bound glypicans bind to lipid-modified Wnts and enable their transport by moving along the membrane and relaying Wnts to other HSPGs and finally to Fz receptors present on receiving cells. **(D)** Exosomes, small vesicular structures loaded with Wnts both free and Evi bound form in the MVBs and released outside cells, further carrying them to receiving cells. **(E)** Cytonemes, cell membrane extensions directed by the cytoskeletal reorganization that extend up to several cell distances and carry Wnts along with them to the receiving cells. In some cases, the cytonemes extending from the receiving cells carrying Fz receptors bring receptors to the Wnt-producing cells.

Once released by the cells, Wnt proteins travel in the extracellular space to activate signaling up to several cell distances. However, the lipid-modification of Wnts renders them hydrophobic, which makes it difficult to envisage their extracellular movement with simple models such as free diffusion. Over the past two decades, several modes for extracellular Wnt travel assisted by a number of carrier molecules have been identified. These carriers interact with Wnt proteins and mask their hydrophobic domains aiding their travel in the aqueous extracellular space. Here, we summarize some of the Wnt carriers, focusing mostly on studies with *Drosophila* Wg (Figure 3). For further detailed information, readers are directed to the following reviews: Takada et al. (2017) and Routledge and Scholpp (2019).

One proposed mechanism for the extracellular transport of Wnt is via their oligomerization (as micelles) which is also observed for other secreted hydrophobic proteins, like Hedgehog (Chen et al., 2004). This oligomerization is believed to shield their hydrophobic regions. However, a recent study in flies using a combination of GFP and Myc tagged Wg protein revealed that blocking the movement of GFP labeled Wg did not affect the spreading of Myc labeled-Wg suggesting that Wnts may not travel in the extracellular space as oligomers or micelles (McGough et al., 2020). This further supports the possibility that Wnt proteins would require specific carrier molecules for their extracellular travel.

*In vitro* studies in *Drosophila* identified a lipocalin family protein known as Swim (Secreted Wingless Interacting Molecule), which binds Wg (Figure 3A). Through this interaction, Swim proteins not only aid the long-range extracellular travel of Wg but also stabilize its signaling activity by maintaining the solubility of Wg (Mulligan et al., 2012). RNAi-mediated depletion of *Swim* expression *in vivo* impedes the stability of Wg and hence its distribution over the long-range. In contrast to this, the *Swim* mutant did not show Wg spreading or signaling defects, indicating that Swim could be dispensable for Wg transport (McGough et al., 2020). Thus, further analysis is required to ascertain their role in Wg transport and to identify possible redundancies between lipocalins.

Besides Swim, lipoprotein particles (LPP) can also interact with lipid-modified Wnts and act as a vehicle for their extracellular transport (Figure 3B). This has been best demonstrated with the mammalian Wnt proteins. For instance, an earlier study showed that active lipid-modified Wnt3a molecules secreted by cultured mammalian cells are associated with the LPP and Wnt3a secretion is regulated by high-density lipoproteins (Neumann et al., 2009). Furthermore, an *in vivo* study using mouse hindbrain showed that Wnt5A is released and transported via LPP in the cerebrospinal fluid (Kaiser et al., 2019). However, secretion of *Drosophila* Wg with LPP remains controversial. On one hand, Wg was shown to colocalize with lipophorins (similar to mammalian lipoprotein particles) in the *Drosophila* wing epithelium, and depletion of lipophorins led to the impaired spreading of Wg and concomitant defects in long-range signaling (Panáková et al., 2005). On the other hand, restricting the spreading of GFP-tagged Wg did not affect the distribution of lipophorins in the wing epithelium (McGough et al., 2020), arguing against the movement of Wnt in association with LPP. It is possible that the role of LPP in the extracellular movement of Wnt proteins is context-dependent rather than a universal mechanism.

An alternative mechanism for the transport of Wnt proteins is through their interaction with heparan sulfate proteoglycans (HSPGs) (Reichsman et al., 1996; Mii and Takada, 2020; Figure 3C). For example, Dally, Dally-like proteins (Dlp) are membrane-associated HSPGs that can interact and stabilize Wnt/Wg (Baeg et al., 2001; Han et al., 2005; Yan et al., 2009). Besides, secreted HSPG-Perlecan/Trol was also shown to stabilize Wg in the extracellular space at the *Drosophila* neuromuscular junctions (NMJ) (Kamimura et al., 2013). A recent structural analysis of Wg and Dlp interactions identified regions in Dlp where the Wg palmitoleate group can be accommodated, which stabilizes Wg for extracellular movement (McGough et al., 2020). Similar in function to Dlp, another conserved protein, Reggie-1/Flotillin-2 has been reported to enhance long-range spreading Wg and thus increasing its long-range signaling activity (Katanaev et al., 2008).

A different carrier reported for extracellular Wg transport is exosomes (Figure 3D). These are extracellular vesicles of 40–100 nm diameter formed in the multivesicular bodies (MVB) and released upon the fusion of MVBs with the plasma membrane (Gross and Boutros, 2013; Hessvik and Llorente, 2018). Initial studies in the *Drosophila* wing epithelium suggested that “argosomes,” which were believed to be extracellular vesicles, could carry Wg in the extracellular space (Greco et al., 2001). Later, Wg was shown to cross NMJ on Evi positive exosomes (Korkut et al., 2009; Koles et al., 2012). Further studies have shown that Wnt proteins are released on exosomes in mammalian cells as well as in cultured *Drosophila* cells and epithelial tissues (Gross et al., 2012; Beckett et al., 2013). However, unlike the NMJ, Wg and Evi are not released on the exosome together and most likely separate in the late endosomal compartments of the producing cell (Gross et al., 2012; Beckett et al., 2013).

Specialized cellular actin-rich filopodia like cell protrusions called cytonemes (Ramírez-Weber and Kornberg, 1999) have also been reported to assist in the long-range activity of Wnt proteins (Stanganello et al., 2015), along with other signaling ligands, for example, Dpp (Roy et al., 2014) and Hedgehog (Bischoff et al., 2013; Figure 3E). However, the mechanisms by which they mediate long-range Wnt signaling are context-dependent. For example, in vertebrates, cytonemes have been shown to carry Wnt ligands from the source to the target cell (Holzer et al., 2012; Stanganello et al., 2015; Mattes et al., 2018). Conversely, invertebrate tissue like *Drosophila* myoblasts extend cytonemes from the receiving cells (bearing Fz receptors) to the producing cells in order to trap Wg from the wing imaginal disc (Huang and Kornberg, 2015).

In summary, the hydrophobic Wnt ligands employ multiple carrier molecules and mechanisms for their long-range travel. Interestingly, some of these mechanisms operate simultaneously for a particular Wnt ligand in the same tissue, while others are context-dependent (Figure 3). Consequently, an interesting question is whether a proportion of Wnt ligands are released using a particular carrier molecule and if the signaling readouts are due to the combinatorial effect of their range and signaling abilities. Furthermore, the regulatory mechanisms which modulate Wnt trafficking for their loading on different extracellular vehicles remain poorly understood.

## Range of Wnt Protein Activity

Whether Wnts act as long-range signaling molecules has been a long-debated question and the exact range of their action is not known. In vertebrates, the evidence for a long-range action of Wnt proteins is mainly indirect. This is based largely on the observation of a broad expression of Wnt target genes in developing tissues, generated by Wnt proteins secreted from a localized source, for example, during early embryogenesis and limb development (Gavin et al., 1990; Kiecker and Niehrs, 2001; Aulehla et al., 2003, 2007; Gao et al., 2011). Direct visualization of a few tagged Wnt proteins also suggested a short-range mode of action. For example, analysis of an exogenously tagged *Xenopus* Wnt8 in the embryo showed short-range and membrane-associated distribution (Mii and Taira, 2009; Mii et al., 2017). The secreted Wnt8 was associated either with the extracellular inhibitor sFRP, forming a non-functional complex or with *N-*sulfo-rich heparan sulfate as a signaling active complex (Mii et al., 2017). Similar approaches to analyze Wnt3 in the mouse intestinal crypts also showed a short-range distribution mediated via lateral transcytosis (Farin et al., 2016). However, highly sensitive and quantitative imaging techniques, for example, fluorescence correlation spectroscopy (FCS) and fluorescence decay after photoconversion (FDAP), which measure the rate of diffusion, have allowed better analysis of the extracellular movement of Wnt proteins. Employing these techniques to analyze the dispersal dynamics of tagged XWnt8 protein, a recent study showed that extracellular XWnt8 is bound to the cell surface as well as exists in a freely diffusing form (Mii et al., 2021). These two forms of XWnt8 were suggested to be exchangeable, which could facilitate the long-range graded distribution of the protein. Moreover, both short-range and long-range distribution of fluorescently tagged zebrafish Wnt8a has been observed, which was shown to be via cytonemes (Luz et al., 2014; Stanganello et al., 2015).

Gradients of Wnts in invertebrates, such as *C. elegans* have also been observed. Single-molecule fluorescence in situ hybridization (smFISH) showed localized expression of several Wnt genes in *C. elegans* (Harterink et al., 2011a). Later, analysis of an endogenously tagged Wnt-homolog EGL-20, using fluorescence recovery after photobleaching (FRAP), showed a long-range spreading. Moreover, blocking the spreading of tagged Egl-20, using morphotrap, led to defects in neuroblast migration, indicating the functional importance of Wnt spreading (Pani and Goldstein, 2018). Interestingly, this long-range dispersal of Egl-20 is believed to be via free ligand diffusion and the role of any extracellular carrier proteins is unclear. Besides, a recent study analyzed the gradient-dependent function of another *C. elegans* Wnt, Lin-44. It was shown that while the long-range Lin-44 gradient is required for neurite migration and cell fate specification, it was dispensable for neurite pruning, as it remained unaffected by tethering endogenous Lin-44 to the membrane (Lu and Mizumoto, 2019). Thus, the requirement of Wnt protein gradients in developmental processes is context-dependent.

One of the best-known examples for long-range action of Wnt protein, and interestingly also the strongest contradiction has been observed with the *Drosophila* Wg protein, which is the main ligand for canonical Wnt signaling. Using highly efficient antibodies and a labeling method to detect the extracellular proteins, earlier studies have demonstrated the presence of a long-range extracellular Wg gradient in the wing imaginal discs, extending on both sides of the stripe of secreting cells residing at the dorsal-ventral (DV) boundary (Strigini and Cohen, 2000). Besides this, a graded expression of Wg target genes, for example, *Distal-less (Dll)* is also detected across the wing disc primordium (Struhl and Basler, 1993; Zecca et al., 1996; Neumann and Cohen, 1997; Cadigan et al., 1998). Moreover, clonal expression of Wg, but not a membrane-tethered Wg protein led to the activation of signaling in cells located distally at a distance (Zecca et al., 1996). Further analysis has shown that Wg can reach at least till 11 cell distances from the DV boundary to directly activate gene expression (Cadigan et al., 1998; Chaudhary et al., 2019), highlighting a direct long-range action of Wg in the developing wing epithelium. However, in the *Drosophila* embryo, the range of Wg is restricted to only a few adjacent cells from the producing cells (DiNardo et al., 1988; Martinez Arias et al., 1988; Vincent and Lawrence, 1994), indicating that the functional range of Wg is contextual.

The paradigm of long-range Wg signaling has been further complicated by the dynamic changes in the Wg expression during the development of wing discs. Studies have shown that Wg expression pattern changes gradually from a broader expression in the entire wing pouch region during early larval stages to a narrow stripe of cells at the DV boundary, at later stages (Couso et al., 1993; Williams et al., 1993; Ng et al., 1996; Rulifson et al., 1996; García-García et al., 1999; Alexandre et al., 2014). This led to the conception of a different model whereby the broader expression of Wg rather than its secretion and the long-range gradient is believed to generate the graded expression of the target genes. This model has been also supported by studies on vertebrate limb development, where a broad expression of Wnt5a was shown to regulate mesodermal patterning and the PCP pathway (Parr et al., 1993; Yamaguchi et al., 1999; Fisher et al., 2008; Gao et al., 2011, 2018). The model was further tested in flies by replacing the endogenous Wg with a membrane-tethered Neurotactin tagged Wg (NRT-Wg) fusion protein, which remained restricted on the surface of producing cells and thus presumed to act in a juxtacrine manner. Surprisingly, the *NRT-wg* flies emerged as normal appearing adults although with smaller but normally patterned wings and with developmental delay (Alexandre et al., 2014). Also, the expression pattern of long-range target gene *Dll and fz3* in *NRT-wg* discs was comparable to that of the wildtype (Alexandre et al., 2014). This indicates that the long-range Wg spreading may not be necessary for broad expression of target genes, which once activated could be further maintained in a ligand-independent manner. However, the mode of action of NRT-Wg and Wg appears to be different, as it was recently shown that downregulation of the early broader expression of *NRT-wg* but not the endogenous *wg* led to the reduction in target gene expression in the receiving cells (Chaudhary et al., 2019). This suggests that a direct long-range effect of Wg, rather than a prior expression in the receiving cell, is required for a broad expression of target genes.

In any case, the observation of a broader gene expression in the absence of a long-range gradient has revealed that there are compensatory mechanisms that could allow maintenance of target gene expression. This could provide developmental robustness to the growing tissues. However, these mechanisms may work in a tissue-specific manner. For instance, recent studies have shown that the *NRT-wg* flies do have other defects, for example in the proximo-distal patterning in the *Drosophila* renal tube (Beaven and Denholm, 2018) and patterning of *Drosophila* intestinal epithelial and muscle tissues (Tian et al., 2019), which appears to be dependent on the long-range spreading of Wg.

## Feedback Regulation for Robust Wnt Signaling in Developing Tissues

**FIGURE 4:**
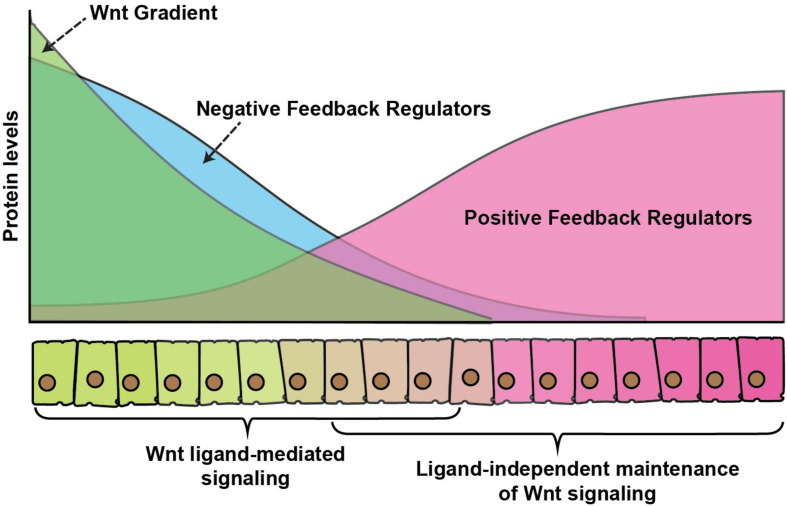
Wnt gradient and feedback regulators. A graphical representation of feedback regulation of Wnt signaling. Green color represents secreted Wnt protein gradient over the receiving cells. Blue color represents expression of negative feedback regulators which is higher in cells exposed to high Wnt levels, whereas positive feedback regulators (red) are higher in cells with exposure to low-levels or no Wnt ligands. The signaling activity is regulated by a combinatorial effect of direct ligand-mediated signaling, the dampening effect of the negative feedback regulators and enhancement of ligand or maintenance of signaling in the absence of ligand by the positive feedback regulators.

It is somewhat easier to envisage how a morphogen gradient could directly pattern an unchanging field of cells, however, in rapidly developing tissues, cells are rarely stationary. Thus, a challenging problem in developing tissues is how cells balance signaling levels, while experiencing a constant change in the levels of extracellular ligands. It is now well-accepted that numerous signaling pathways use positive and negative feedback mechanisms to ensure balanced signaling and maintain developmental robustness. Positive feedback is required for the amplification of a weak signal whereas negative feedback dampens signaling. Moreover, signaling pathways regulate the expression of these feedback regulators, whereby the expression of positive regulators is generally suppressed and negative regulators are activated by high-level signaling (Figure 4).

The canonical Wnt signaling pathway has highly diverse and complex feedback regulations, which allows it to modulate signaling in various developmental contexts. For example, in vertebrates, a Wnt signaling induced protein Axin2 acts as a cytoplasmic inhibitor of Wnt signaling (Jho et al., 2002; Lustig et al., 2002), which dampens excess signaling. Similarly, another Wnt target Naked cuticle inhibits Wnt signaling by interacting with the cytoplasmic protein Dsh. While Axin mediated feedback regulation is not observed in organisms, for example, *Drosophila*, Naked cuticle on the other hand is highly conserved (Rousset et al., 2001; Wharton et al., 2001). Besides this, secreted inhibitors of Wnt proteins are also expressed by Wnt signaling, for example, a highly conserved extracellular protein Notum binds and deacetylates Wnts which reduces their signaling activity (Kakugawa et al., 2015).

A common mechanism of feedback regulation is by modulating signaling at the level of surface receptors. For example, in vertebrate a set of Wnt target genes, including Dickkopf-1, *Rnf43* and *Znrf3* inhibits Wnt signaling at the receptor level by either interacting with the co-receptor LRP5/6 (Glinka et al., 1998; Bafico et al., 2001; Semënov et al., 2001; Niida et al., 2004) or reducing the receptor levels at the membrane by increasing their ubiquitination-mediated internalization (Hao et al., 2012; Koo et al., 2012). In *Drosophila*, the Fz receptors are part of both positive and negative feedback regulations. For instance, the expression of *fz3* receptor is activated by the canonical Wg signaling and loss of *fz3* was shown to rescue the morphological defects in *wg* hypomorphic mutants, suggesting that it acts as a negative regulator of the pathway (Sato et al., 1999). In contrast to Fz3, the Fz2 receptor acts redundantly with the Fz1 receptor to activate canonical signaling and it is transcriptionally repressed by Wg signaling (Bhat, 1998; Kennerdell and Carthew, 1998; Bhanot et al., 1999; Chen and Struhl, 1999; Müller et al., 1999). In the developing wing imaginal discs, the expression of Fz3 is graded with higher levels near the DV boundary, whereas the Fz2 receptor is expressed in a reversely graded manner (Figure 4). Similarly, the Fz co-receptor Arrow, which is required for the canonical pathway is also transcriptionally repressed by the signaling (Wehrli et al., 2000). Together, these receptors can modulate the variations in the Wg signaling activities and provide developmental robustness (Cadigan et al., 1998; Chaudhary et al., 2019).

Another mechanism to achieve this developmental robustness could work at the level of target gene expression. While it is commonly understood that the expression of target genes depends on the signal activation directly by the ligand-receptor complexes, studies have also suggested that once activated, expression of certain target genes can persist in the absence of the extracellular ligand, which is generally referred to as “cellular memory.” For example, the expression of Wg target gene in the *Drosophila* leg imaginal disc is activated by the transient action of Wg and Dpp while at later stages of development this expression is maintained in the absence of Wg via cis-regulatory elements (Galindo et al., 2002; Estella et al., 2008). Furthermore, in the *Drosophila* wing epithelium removal of Wg at later stages of development showed persisted expression of low-threshold targets, Dll and Vg (Piddini and Vincent, 2009). As discussed above, in the wing imaginal discs expression of these target genes could also be seen beyond the observable range of membrane-tethered NRT-Wg (Alexandre et al., 2014). However, unlike the leg discs, the cis-regulatory elements are not believed to be involved in this maintenance in the wing discs (Estella et al., 2008). Therefore, other alternative mechanisms may also be involved in the maintenance of signaling in the absence of the ligand.

For example, one possible mechanism could be via ligand-independent signaling by the Frizzled receptors. A recent study showed that the apparent normal appearing long-range expression of Wg target genes in the membrane-tethered NRT-Wg expressing wing imaginal discs, is mediated via the Fz2 receptors (Alexandre et al., 2014; Chaudhary et al., 2019). Therefore, loss of Fz2 in the NRT-Wg discs showed a reduction in the range of low-threshold target gene expression and a concomitant reduction in cell survival (Chaudhary et al., 2019).

## Mechanisms of Ligand-Independent Signaling by Frizzled Receptors

**FIGURE 5:**
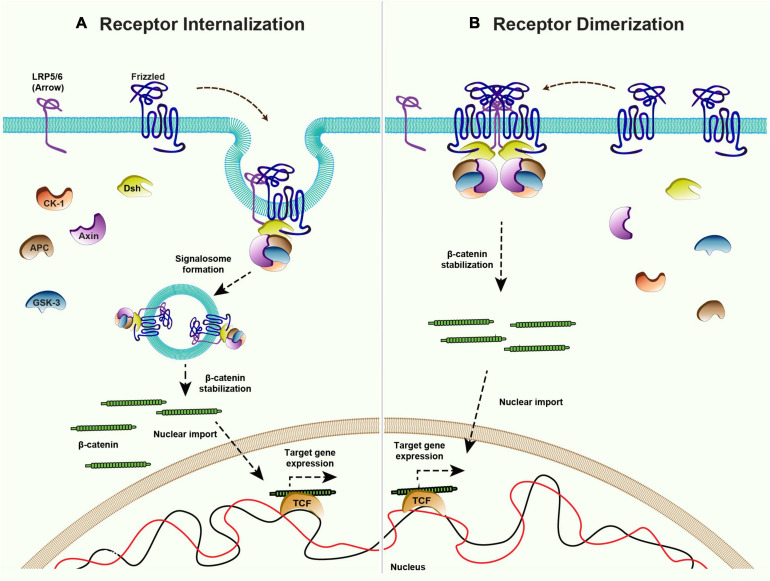
Mechanisms of ligand-independent Wnt signaling. Canonical Wnt signaling is maintained in the absence of ligands via receptor internalization and dimerization. **(A)** Internalization of the Frizzled receptors and co-receptor LRP5/6 could lead to inhibition of the destruction complex and therefore activation of the downstream β-catenin signaling. **(B)** Higher levels of Frizzled receptor in absence of ligand allows the dimerization of Frizzleds which further recruits Dishevelled and rest of the components of the destruction complex to stabilize β-catenin and activate the target gene expression.

While the mechanism of ligand-independent receptor activation of canonical signaling remains poorly understood, some insight came from the overexpression studies with vertebrate Fz receptors. For example, the overexpression of rat and *Xenopus* Fz receptors was shown to be sufficient for the activation of Wnt target gene expression in *Xenopus* embryo (Yang-Snyder et al., 1996; Umbhauer et al., 2000). Studies have shown that the overexpression of *Xenopus* Fz3 receptor leads to its dimerization, which can mediate ligand-independent signal activation (Carron et al., 2003). Furthermore, as mentioned above, the activation of canonical signaling is triggered by the formation of signalosome complexes (Bilic et al., 2007; Gammons et al., 2016) and Wnt proteins are believed to act as a mediator of Fz and LRP5/6 oligomerization. In line with this, the oligomerization of the Fz receptor and LRP5/6 to form signalosome and internalization was shown to be sufficient to activate ligand-independent β-catenin signaling in APC mutant prostate cancer cells (Hua et al., 2018; Saito-Diaz et al., 2018). Therefore, an increase in the levels of the Fz receptors may be sufficient for the formation of a signalosome complex and the activation of signaling in a ligand-independent manner (Figure 5).

The family of Frizzled receptors have several members but do all the receptors possess the ability to maintain canonical Wnt signaling? As mentioned above Fz2 acts redundantly along with Fz1 to activate canonical Wg signaling in *Drosophila* wing epithelium, but loss of Fz2 and not Fz1 affects the maintenance of target gene expression (Chaudhary et al., 2019). Similarly, overexpression XFz3 but not the XFz7 was able to activate signaling without Wnt ligand in *Xenopus* embryo (Yang-Snyder et al., 1996; Umbhauer et al., 2000). These findings suggest that maintenance of signaling is specific rather than being a general characteristic of Fz receptors. A major hindrance in understanding the mechanism is the lack of structural information regarding the activation of Fz receptors. Until now, less attention has been given to Wnt-independent activation of Fz receptors and the exact mechanism behind such activation remains a mystery.

## Conclusion

The processes and mechanisms related to Wnts and their signaling have been extensively studied due to their involvement in numerous cellular processes covering various developmental aspects. Using different model organisms, studies reviewed here have shown that the mechanisms regulating steps like Wnt protein expression and modification, their polarized secretion and modes of extracellular transport, their reception and signaling activity in the receiving cells, are diverse and context-dependent. However, the identification of specific and well-conserved regulatory proteins involved at various steps also indicate that the Wnt-related processes are tightly regulated.

An unanswered question is how Wnt proteins utilize these multiple non-redundant functional routes, to finally converge at a particular signaling outcome and fate of the cell. Further, fine dissection of each route will be necessary to ascertain its functional specificity and to identify the regulatory processes directing the Wnt protein toward a particular functional route. In parallel to this, studies focusing on analyzing the contribution of feedback regulations and ligand-independent signaling mechanisms will provide a better understanding of the processes facilitating developmental robustness.

## Author Contributions

SM, SH, and VC conceptualized and wrote the manuscript. SM and SH contributed equally. All authors read and approved the final manuscript.

## Conflict of Interest

The authors declare that the research was conducted in the absence of any commercial or financial relationships that could be construed as a potential conflict of interest.

## Publisher’s Note

All claims expressed in this article are solely those of the authors and do not necessarily represent those of their affiliated organizations, or those of the publisher, the editors and the reviewers. Any product that may be evaluated in this article, or claim that may be made by its manufacturer, is not guaranteed or endorsed by the publisher.

## Footnotes

1
http://wnt.stanford.edu
